# Tackling psychosocial maladjustment in Parkinson’s disease patients following subthalamic deep-brain stimulation: A randomised clinical trial

**DOI:** 10.1371/journal.pone.0174512

**Published:** 2017-04-11

**Authors:** Joao Flores Alves Dos Santos, Sophie Tezenas du Montcel, Marcella Gargiulo, Cecile Behar, Sébastien Montel, Thierry Hergueta, Soledad Navarro, Hayat Belaid, Pauline Cloitre, Carine Karachi, Luc Mallet, Marie-Laure Welter

**Affiliations:** 1 Inserm U 1127, CNRS UMR 7225, Sorbonne Universités, UPMC Univ Paris 06 UMR S 1127, Institut du Cerveau et de la Moelle épinière, ICM, Paris, France; 2 AP-HP, Personalised Neurology & Psychiatry University Department, Hôpitaux Universitaires Henri Mondor–Albert Chenevier, Université Paris Est Créteil, Créteil, France; 3 Department of Mental Health and Psychiatry, Geneva University Hospital, University of Geneva, Geneva, Switzerland; 4 AP-HP, Groupe Hospitalier Pitie-Salpêtrière, Biostatistics Unit and Clinical Research Unit, Paris, France; 5 Sorbonne Universités, UPMC Univ Paris 06 UMR_S1136, and INSERM UMR_S 1136, Institut Pierre Louis d’Epidémiologie et de Santé Publique, Paris, France; 6 AP-HP, Hôpital de la Salpêtrière, Genetic Department, Paris, France; 7 AP-HP, Hôpital de la Salpêtrière, Neurology Department, Paris, France; 8 AP-HP, Hôpital de la Salpêtrière, Centre d'Investigation Clinique, Paris, France; 9 AP-HP, Hôpital de la Salpêtrière, Neurosurgery Department, Paris, France; University of Toronto, CANADA

## Abstract

**Background:**

Subthalamic nucleus deep brain stimulation (STN-DBS) is an effective treatment for the motor and non-motor signs of Parkinson’s disease (PD), however, psychological disorders and social maladjustment have been reported in about one third of patients after STN-DBS. We propose here a perioperative psychoeducation programme to limit such social and familial disruption.

**Methods:**

Nineteen PD patients and carers were included in a randomised single blind study. Social adjustment scale (SAS) scores from patients and carers that received the psychoeducation programme (*n =* 9) were compared, both 1 and 2 years after surgery, with patients and carers with usual care (*n =* 10). Depression, anxiety, cognitive status, apathy, coping, parkinsonian disability, quality-of-life, carers’ anxiety and burden were also analysed.

**Results:**

Seventeen patients completed the study, 2 were excluded from the final analysis because of adverse events. At 1 year, 2/7 patients with psychoeducation and 8/10 with usual care had an aggravation in at least one domain of the SAS (p = .058). At 2 years, only 1 patient with psychoeducation suffered persistent aggravated social adjustment as compared to 8 patients with usual care (p = .015). At 1 year, anxiety, depression and instrumental coping ratings improved more in the psychoeducation than in the usual care group (p = .038, p = .050 and p = .050, respectively). No significant differences were found between groups for quality of life, cognitive status, apathy or motor disability.

**Conclusions:**

Our results suggest that a perioperative psychoeducation programme prevents social maladjustment in PD patients following STN-DBS and improves anxiety and depression compared to usual care. These preliminary data need to be confirmed in larger studies.

## Introduction

Parkinson's disease (PD) is a progressive neurodegenerative disorder classically characterised by motor signs, (i.e. tremor, bradykinesia, rigidity, gait and balance disorders) and also non-motor symptoms with cognitive and neuropsychiatric disorders such as depression, apathy and anxiety [1]. For over fifteen years, deep brain stimulation (DBS) of the subthalamic nucleus (STN) has been shown to be an efficient means of improving motor disability while allowing a reduction of dopaminergic drug treatment and levodopa-induced motor complications [2]. Despite this dramatic improvement, previous studies have frequently reported negative psychological outcomes. Indeed, STN-DBS may provoke hypomanic status or impulsivity, which can be improved by the interruption of STN-DBS or by modifying the parameter settings [3–5]. The psychological consequences of STN-DBS are still a subject of debate with some studies reporting an improvement or no change in depression and/or anxiety [6–9] and others an aggravation [10,11]. In addition, changes in self-image and personality traits have also been reported [12,13] with perceived outcome subjectively judged as negative [14]. The latter negative psychological outcomes may result in a reduced improvement in quality of life, but also reduced social and familial adjustment with a paradoxical aggravation in about one third of cases with work disruption, marital or familial discord [7,12,14,15]. This postoperative maladjustment was initially described as a “burden of health” given the patients’ difficulties to return to their “normal” life after surgery [16]. More recently, it has been matched to the “burden of normality” syndrome, initially described within the context of anterior-temporal lobectomy in epilepsy patients [17] who experienced psychological and social complications following the “chronically ill” to the “seizure free” transition. No clinical factor has been clearly identified in relation to the occurrence of postoperative maladjustment, but patients’ realistic vs. unrealistic expectations about the treatment seem to play a central role [18–21]. Psychoeducational interventions have been previously proposed as a method to accompany medical treatments with positive impact on treatment outcome and patients’ psychological adjustment [22–28]. Nevertheless no specific intervention has been advocated to avoid these psychological side effects following STN-DBS. In this prospective randomised controlled study, we report the effects of a psychoeducation programme, specifically designed for PD patients enrolled for STN-DBS and for their respective carers, given their central role on the dyad psychological adjustment after DBS-STN [29].

## Materials and methods

### Participants

**Fig 1:**
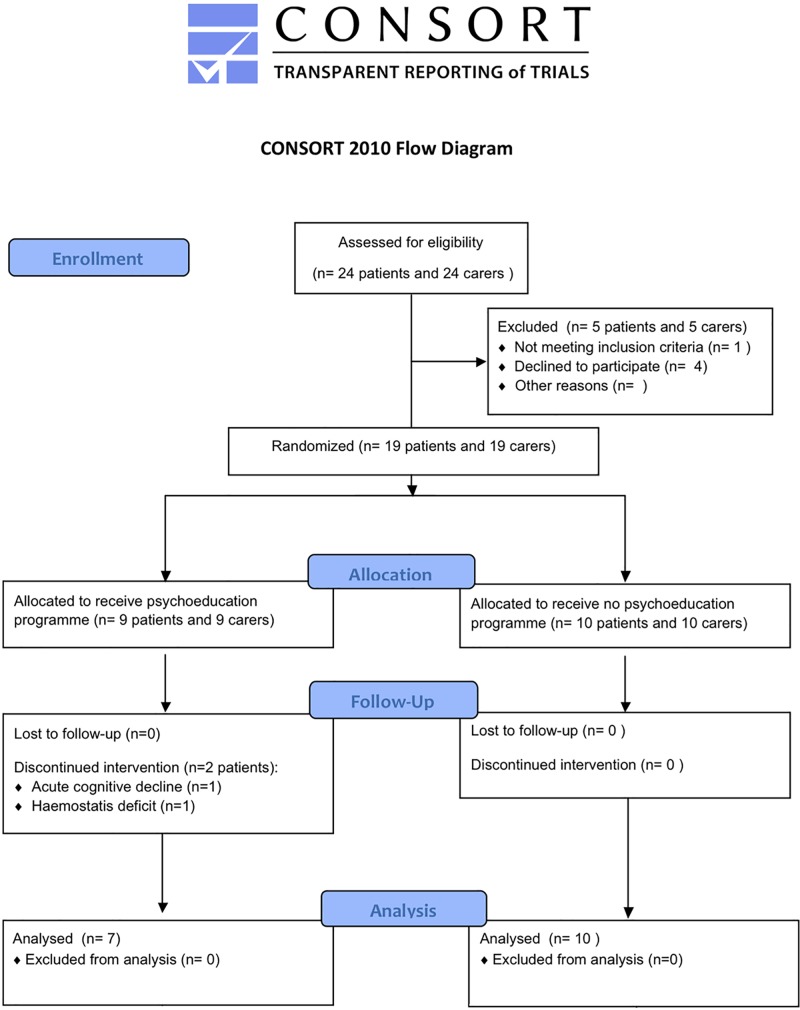
Flow-chart of the study.

Nineteen PD candidates for STN-DBS (4 women and 15 men, median age [Interquartile range] = 60 [52–65] years, median disease duration [Interquartile range] = 9 [8–15] years) and their respective carers (spouses in all cases, 4 men and 15 women, median age [Interquartile range] = 59 [52–65] years) were recruited in this prospective randomised controlled study between February 2008 and September 2011 (APHP Promotion P060101, N° IRCD: 2006-A00230-51, Fig 1). The study was approved by the local ethics committee (CPP Ile-de-France Paris VI, N° CPP: 3–07, date: 24/08/2007), and was registered at the ClinicalTrials.gov website (NCT02554370) after enrolment of participants due to the sponsor’s initial omission. The authors confirm that all ongoing and related trials for this intervention are registered. All participants completed and signed a written information and consent form.

Patient’s inclusion criteria were the inclusion criteria for STN-DBS: diagnosis of idiopathic PD, age between 20 and 70 years old, moderate to severe motor parkinsonian disability (Hoehn & Yahr without levodopa treatment (OFF) score ≥ 2.5) with a good levodopa response (> 50%), disabling levodopa-induced motor complications despite optimal antiparkinsonian drug treatment (Table 1) [30]. Patients with levodopa resistant motor axial signs, dementia (Mattis dementia rating scale < 130) [31], severe psychiatric (psychosis, bipolar disorder, major depressive disorder or severe personality disorder assessed with the Mini International Neuropsychiatric Interview-MINI and Temperament and Character Inventory-TCI) [32,33] or any additional neurological disorders were excluded.

**Table 1:** Biographical and clinical characteristics of the 19 PD patients candidate for STN-DBS.

Subject	Group	Sex	Age (yrs)	Education level	Actual or past occupation	Disease duration (yrs)	Parkinsonian motor disability		LEDD (mg)	MDRS	Past medical history	Psychotropic treatment
							OFF levodopa	ON levodopa				
1	UT	M	51	3	Farmer (FT)	8	21	2	1100	141	Hypercholesterolemia	None
2	PSY	M	44	4	Clerk (PT)	10	48	1	1150	144	Depression, Appendectomy	Alprazolam
3	PSY	M	53	5	Car mechanic (DI)	12	10	2	900	136	Ankle sprain	None
4	PSY	M	44	2	Building contractor (DI)	7	43	16	1000	142	Depression, Hypercholesterolemia	Citalopram
5	UT	M	63	7	Entrepreneur (RE)	13	24	0	900	144	Hypertension, benign pancreatic tumour	Bromazepam
6	UT	W	69	7	Housewife	8	19	4	800	144	Ulcerative colitis	Prazepam
7	UT	W	45	7	Nurse (DI)	8	19	0	450	144	Epilepsy, Thyroid nodule	None
8	PSY	M	70	7	Employee trade (RE)	15	45	15	1350	134	Depression, Melanoma, Lombosciatica	Paroxetine
9	UT	M	60	5	Employee trade (RE)	8	41	4	1600	143	Depression, Hypercholesterolemia, Inguinal hernia surgery	Venlafaxine
10	PSY	W	59	6	Housewife	9	37	3	700	144	Depression, Hypertension, Glaucoma	Seropram, Clonazepam
11	PSY	M	52	4	Driver (FT)	8	69	29	850	136	None	None
12	UT	M	62	7	Computer scientist (RE)	20	29	5	1050	141	Hypertension	None
13	UT	M	64	4	Bank employee (RE)	12	34	1	1250	141	Depression, Prostatic surgery	Seropram, Clonazepam
14	UT	W	70	4	Housewife	15	21	11	1000	137	Hypercholesterolemia	None
15	PSY	M	70	7	Entrepreneur (RE)	8	37	15	1100	127	Inguinal hernia surgery	None
16	PSY	M	67	4	Employee trade (RE)	9	21	5	1200	135	Asthma, Inguinal hernia surgery	None
17	UT	M	61	4	Electrician (RE)	20	29	7	1000	131	Depression, Hypercholesterolemia	Seropram, Alprazolam
18	UT	M	59	6	Printer (DI)	21	54	13	1050	138	None	Clonazepam
19	PSY	M	59	7	Project manager (FT)	7	26	3	1150	142	Myocardial infarction	None

UT: usual treatment alone; PSY: psychoeducation; M: men; W: women; OFF: without levodopa treatment; ON: with levodopa treatment; LLEDD: levodopa-equivalent daily dosage; FT: full time; PT: part time, DI: disability; RE: retired

### Study design

We hypothesised that our psychoeducational programme, focussing on patients’ and carers’ expectations, should decrease the occurrence of social maladjustment in PD patients 1 year after surgery (*hypothesis 1*). We also looked to see whether changes in social adjustment persist 2 years after surgery and whether they are beneficial for carers (*hypothesis 2*). After inclusion, the patients’ social adjustment was assessed with the Social Adjustment Scale (SAS) [34]. This scale comprises 6 domains: work, social life, family, couple, children and global social adjustment, evaluated during an interview with 6 to 14 items per domain. At the end of the interview, each domain was rated from 1 (excellent) to 7 (very severe maladjustment) by the investigator, using the ‘global evaluation part’ of the SAS. Eligible patients (and their carers) were randomly assigned in a 1:1 ratio to one of two groups: one group underwent the psychoeducation programme and usual treatment and the other underwent the usual treatment alone. We used a blocking-scheme and a centralised procedure for randomisation, with stratification according to their preoperative couple SAS domain score (Fig 1).

The main outcome criterion was the “couple” SAS domain score obtained 1 year after surgery in PD patients. The SAS interview was videorecorded and scored (all subdomains: work, social life, family, couple, children and global social adjustment) by an independent evaluator blind to the treatment group. In line with our previous studies [7,12], within this research programme we expected that 2/3 patients in the psychoeducation group would have full socio-familial adjustment one year after surgery and none in the usual treatment group. Given these assumptions (normal scores test, two-tailed test, alpha = 5%, exact test), having 10 patients in each group provides a power of 70%.

### The psychoeducation programme

**Fig 2:**
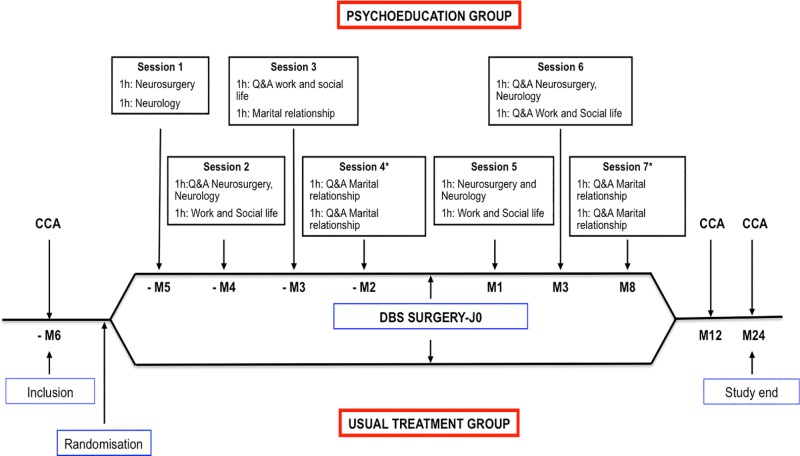
Randomised, single-blind, parallel design of the study. Patients were included and randomised 6 months before surgery for the psychoeducation programme (above) or usual treatment (below). CCA: Complete clinical assessment; Q&A: Question and Answer; * Individual session.

Psychoeducational intervention was centred on information transmission, identification and clarification of patients’ and carers’ expectations and coping preparation, targeting not only the restoration of patients’ sense of control during the entire treatment but also the adjustment of maladaptive thinking processes. The psychoeducation programme focused on 3 main domains: *first—*neurosurgical procedure and neurological outcome, which was performed by the neurosurgeon and neurologist; *second—*social life impact including work, social and familial relationship, performed by the neurologist and psychologists; *third–*the couple relationship, with each couple being interviewed individually for one hour by a neurologist, psychiatrist and psychologists, in a joint session. The programme consisted of 7 two-hour sessions, which were attended by groups of 3 to 4 couples per session, with the exception of the *third* domain–the couple relationship—where sessions were performed for each couple individually. Each domain was tackled in a two-way manner, first information was provided by the investigators and second they encouraged the discussion to be about the patients’ and carers’ expectations. Each successive session started with a “Question and Answer” period about the domain previously discussed and then, once again, information/education on the next domain was given. Four sessions were performed 5, 4, 3 and 2 months before surgery and 3 sessions 1, 3 and 8 months after surgery (Fig 2). Specifically, preoperative sessions started with a 15–20 minutes educational block performed by investigators aimed at information transmission, regarding each individual domain, using a rich multimedia slideshow (S1 File). No media resources were used during the sessions after surgery (Fig 2).

### Clinical assessment

Patients and carers were evaluated 6 months before and 1 and 2 years after surgery (Table 2).

**Table 2:** Changes in social adjustment, quality of life, anxiety, coping strategies, cognitive processes/abilities/functions and parkinsonian disability, both 1 and 2 years after surgery.

		Psychoeducation group			Usual treatment group		
		Before surgery	Change with STN-DBS		Before surgery	Change with STN-DBS	
			1 year	2 years		1 year	2 years
**Social Adjustment Scale (SAS)**							
	Work	3 [2;4]	0 [-3;0]	-1 [-3.5;-0.5]	3 [2;3]	0 [0;1]	1 [0;2]
	Social life	3 [2;4]	-1 [-1;0]	-1 [-2;0]	3 [2;4]	-0.5 [-1;1]	0 [0;1]
	Family	2 [2;3]	-0.5 [-1;0]	-1 [-1;0]	2 [2;2]	0 [0;0]	**0 [0;1]**^**a**^
	Couple	3 [2;4]	0 [-2;1]	0 [-2;0]	3 [2;4]	0 [-1;0]	0 [-1;0]
	Children	2 [2;3]	-1 [-1;0]	-1 [-1;-1]	2 [2;3]	-0.5 [-1;0]	-1 [-1;0]
	Global	3 [2;3]	0 [-2;0]	-1 [-1;0]	3 [3;3]	0 [0;1]	0 [-1;0]
**Quality of life**							
	PDQ_39 SI	35 [31;53]	-12.6 [-15;-10]	-11.7 [-14;-10]	35 [32;38]	-2.8 [-12;4]	-1.0 [-11;2]
	Whoqol_26	64 [53;82]	-1 [-9;10]	-32 [-32;15]	83 [74;93]	-15.5 [-27.5;-5.5]	-10 [-16;-1]
**Depression (MADRS)**		10 [7;18]	**-6 [-15;-2] (n = 9)**	**-8 [-13;-1]**	7 [5;10]	**1 [-2;2]**^**a**^	**2.5 [-2;7]**^**a**^
**Anxiety**							
	STAI State	47 [44;50]	**-6 [-8;-3]**	1 [-6;4]	52 [48;45]	**-0.5 [-5;0]**^**a**^	-1.5 [-6;0]
	STAI Traits	43 [39;52]	2 [-4;2]	0 [-4;2]	47.5 [45;50]	-3.5 [-5;-1]	-2.5 [-4;1]
**Coping (CHIP)**							
	Emotional	32 [23;35]	-1 [-16;5]	-12 [-33;-5]	27 [19;31]	-4.5 [-11;0]	-5 [-12;-5]
	Instrumental	23 [21;31]	**2 [-1;2]**	-4 [-9;1]	31 [25;33]	**-5.5 [-8;-1]**^**a**^	-6 [-8;-3]
	Palliative	20 [19;23]	2 [-3;6]	-1 [-4;8]	21.5 [18;23]	1.5 [-1;4]	-1.5 [-3;7]
	Distraction	28 [18;29]	2 [-8;2]	-5 [-8;-1]	23 [20;29]	-1.5 [-4;0]	-2 [-7;0]
**Apathy (Starkstein)**		6 [4 ;7]	-1 [-3 ;5]	4 [1 ;8]	8 [6 ;9]	4 [2 ;5]	5 [1 ;6]
**Cognition**							
	MDRS	142 [135;144]	-5 [-6;1]	-5 [-8;1]	141 [138;144]	-7 [-7;-5]	-7 [-10;-3]
	FAB	17 [16;18]	0 [-4;1]	-1 [;5;0]	16.5 [16;17]	-0.5 [-3;0]	-2 [-3;-1]
**Parkinsonian disability (UPDRS)**							
	Part I_Mental	1 [0;5]	-1 [-5;1]	1 [;4;1]	1.5 [1;2]	1 [0;3]	0 [0;1]
	Part II_ADL OFF	19 [15;23]	-9 [-11; -1]	-11 [-15;-5]	18 [16;22]	-5 [-6;-1]	-8 [-14;5]
	Part III ADL ON	5 [2;9]	2 [1;9] (n = 5)	1 [-1;6] (n = 5)	7.5 [3;11]	4 [3;9]	1.5 [0;4]
	Part III_Motor disability OFF	37 [21;45]	-18 [-32;-11]	-26 [-32;-6]	26.5 [21;34]	-16 [-21;-8]	-13 [-19;-2]
	Part III Motor disability ON	3 [2;15]	2 [-1;7]	5 [3;6]	4 [1;7]	6 [5;7]	**10.5 [6;12]**^**a**^
	Part IV_LD complications	8 [6;10]	-7 [-8;-6]	-6 [-8;-2]	9.5 [7;11]	-4 [-11;-2]	-8.5 [-9;-5]
**Antiparkinsonian treatment**							
	LEDD (mg/d)	1130 [803;1160]	-357 [-503;-310]	-310 [-466;-168]	1247 [1129;1404]	-557 [-813;-454]	-480 [-866;-406]
	Dopaminergic agonists (mg/d)	300 [100 ;450]	-50 [-300 ;0]	-100 [-300 ;0]	200 [100 ;300]	-100 [-200 ;0]	-100 [-200 ;-50]
**Outpatient visits**			7 [6;11]	6 [5;6]		8 [7;9]	4.5 [2;7]
**Carers**							
	Burden (Zarit)	30 [12;37]	1 [-5;9]	-5 [-9;4]	19 [9;30]	8 [6;12]	7 [-2;17]
	STAI State	50 [49;51]	-3 [-6;4]	-1 [-2;-1]	49 [49;50]	-4 [-7;2]	0.5 [-1;3]
	STAI Traits	47 [44;49]	0 [-1;4]	-2 [-3;0]	45 [43;47]	-1 [-4;4]	2 [-5;2]

Values are median [Interquartile range]; Change is the difference between the score after surgery with STN-DBS (1 and 2 years) and the score before surgery in the same levodopa treatment condition, Off: without levodopa treatment, On: with levodopa treatment. ADL: Activities of daily living; CHIP: Coping with Health Injuries and Problems; FAB: Frontal assessment battery; LEDD: levodopa-equivalent daily dosage; MADRS: Montgomery and Asberg Depression Rating Scale; MDRS: Mattis Dementia Rating Scale; OFF: without levodopa treatment; ON: with levodopa treatment; PDQ-39 SI: Parkinson’s disease quality of life questionnaire score index; SAS: Social Adjustment Scale; STAI: State Trait Anxiety Inventory; UPDRS: Unified Parkinson’s Disease Rating Scale; Whoqol-26: World Health Organisation Quality of Life. Higher scores indicate worse motor or psychiatric symptoms/traits, quality of life and social adjustment, except for MDRS and FAB for which high scores indicate better cognitive function.

^a^p<0.05 for difference in score changes 1 and 2 years after surgery between patient groups (Mann-Whitney tests for quantitative variables, Fisher Exact tests for qualitative variables)

In PD patients, psychiatric assessment included depressive symptom evaluation with the Montgomery-Asberg Depression Rating Scale (MADRS, range:0–60, with higher scores indicating greater depression severity) [35], anxiety evaluation with the State Trait Anxiety Inventory (STAI state and STAI trait scales, range:20–80, with higher scores indicating greater anxiety) [36]; coping strategies with both the Coping Health Injuries and Problems (CHIP, assessing 4 coping dimensions: distraction; palliative; instrumental and emotional preoccupation, range: 0–40 for each dimension) [37] and Ways of Coping Checklist (WCC, 5 coping domains: problem focus; social support; self blaming; positivism and avoidance) [38]. Quality of life was measured using both the summary index of the Parkinson’s Disease Questionnaire (SI-PDQ-39, 8 dimensions: mobility, activities of daily living, emotional well-being, stigma, social support, cognition, communication and bodily discomfort, range:0–100, lower score indicates better quality of life) [39] and the World Health Organisation Quality of Life survey (WHOQOL-BREF, 4 domains: physical health; psychological health; social relationships and environment) [40].

Neuropsychological evaluation focused on executive functions by using the Mattis Dementia Rating Scale (MDRS, range: 0–144, higher score indicates a better cognitive status) [41] and Frontal Assessment Battery (FAB, range: 0–18, higher scores indicates better cognitive status) [42], and apathy using the Starkstein scale (range: 0–42, with higher scores indicating greater apathy, with a score of 14 used as the pathological cut-off) [43].

Parkinsonian disability was assessed with the Unified Parkinson’s Disease Rating Scale (UPDRS) [44]: Part I–mentation, behaviour and mood; Part II–activities of daily living (ADL); Part III–motor examination; Part IV–levodopa-related motor complications. The ADL score was assessed by patient interview in the ‘off’ and ‘on’ antiparkinsonian drug conditions. Before surgery, motor disability was examined after a 12-hour interruption of antiparkinsonian medication (‘off’ drug) and after administration of a single suprathreshold dose of levodopa (‘on’ drug, usual morning levodopa equivalent dosage + 50 mg). After surgery, motor disability was evaluated in 4 conditions, the same day and in the same order: with STN stimulation after a 12-hour interruption of antiparkinsonian medication (‘on’ stimulation ‘off’ drug); after stimulation was stopped for at least 90 minutes (‘off’ stimulation ‘off’ drug); after administration of the suprathreshold dose of levodopa (‘off’ stimulation and ‘on’ drug); and finally with chronic stimulation and levodopa (‘on’ stimulation ‘on’ drug). The levodopa-equivalent dosage, stimulation parameters settings, and number of outpatient visits were also noted for each visit.

Carers were assessed at 6 months before and 1 and 2 years after surgery. Anxiety was assessed using the STAI and burden using the Zarit Burden Interview-ZBI-22 (ZBI-22, range: 0–88, high score indicates higher burden) [45], that measures subjective perception of burden related to the patients’ functional and behavioural impairments.

All patients were asked to report psychotropic medication changes, medical, psychiatric and psychological consultations in a notebook that was recovered at each visit.

### Statistical assessment

Statistical intention-to-treat analysis was carried out. Data are expressed as median [Interquartile range] or n (%). The changes in scores between baseline (before surgery) and 1 year after surgery, and between baseline and 2 years after surgery, were compared between groups with Mann Whitney tests for quantitative variables and Fisher Exact tests for qualitative variables, with the SAS 9.2 statistical package (SAS Institute Inc.). For the SAS, a one point change in a domain was considered as an improvement if negative and an aggravation if positive. All reported *p*-values are two-tailed with a type I error rate considered statistically significant for 5% and below.

## Results

Nine PD patients (1 woman and 8 men, median age [Interquartile range] = 60 [52–65] years, median disease duration [Interquartile range] = 9 [8–15] years) and their carers (median age [Interquartile range] = 59 [59–64] years) were randomised into the psychoeducation programme group and 10 patients (3 women and 7 men, median age [Interquartile range] = 60 [52–65] years, median disease duration [Interquartile range] = 9 [8–15] years) and their carers (median age [Interquartile range] = 57 [48–63] years) into the usual treatment group (Fig 1). After randomisation, 2 patients (P11 and P15) from the psychoeducation group presented adverse events that prevented surgery (preoperative cognitive decline, haemostasis deficit). Finally, 17 patients (psychoeducation, *n =* 7; usual treatment, *n =* 10) and carers were tested before (baseline), 1 and 2 years after surgery.

### Effect of the psychoeducation programme on the social adjustment after STN-DBS

**Fig 3:**
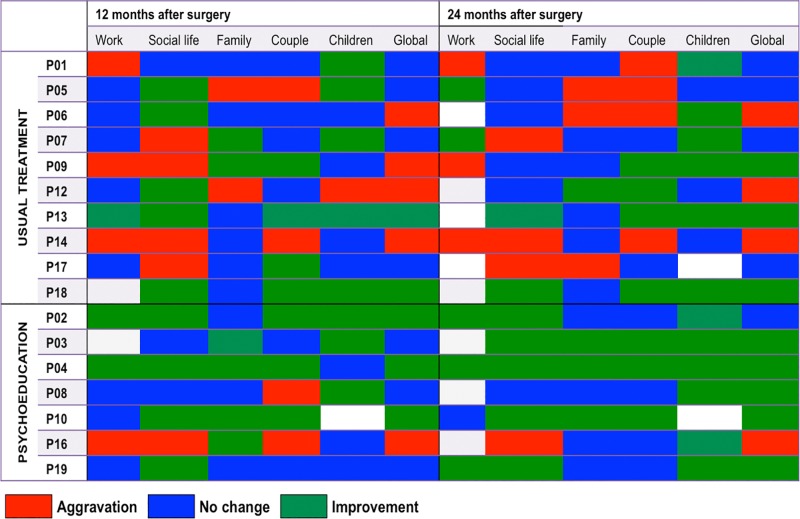
Social adjustment modifications at 1 and 2 years after STN-DBS. Changes in the different domains of the Social Adjustment Scale (SAS) 1 and 2 years after surgery in PD patients were operated for STN-DBS without (above) and with (below) the psychoeducation programme. Aggravation is defined as an increase of ≥1 point of the SAS domain, improvement is defined by a decrease of ≥ 1 point of the SAS domain, in comparison to before the surgery.

One year after surgery, the “couple” subdomain had worsened in 2 patients in both psychoeducation (P08 and P16) and usual treatment groups (P05 and P14), with no significant differences between groups (*p* = .65). Moreover, 2 out of 7 patients in the psychoeducation group (P8 and P16) and 8 out of 10 patients in the usual treatment group (P01, P05, P06, P07, P09, P12, P14 and P17) showed an aggravation of at least one of the social adjustment subdomains (*p* = .058, Fig 3). Two years after surgery, social maladjustment persisted in 8 out of 10 patients of the usual treatment group whereas only 1 patient in the psychoeducation group showed persistent aggravation (P16) (*p* = .015, Fig 3); 3 out of 7 patients in the psychoeducation group showed an improvement in the ‘family’ score whereas, for the same measure, an aggravation was found in 3 out of 10 patients in the usual treatment group (*p* = .043, Fig 3).

### Effects of psychoeducation programme on psychiatric symptoms and quality of life

One year after surgery, patients that participated in the psychoeducation programme showed a significantly greater decrease in depression (*p* = .050) and anxiety scores (*p* = .038), with an improvement in instrumental coping (*p* = .038). Two years after surgery, patients in the psychoeducation programme showed a greater decrease in depression score (*p* = .041) in comparison to patients following the usual treatment (Table 2).

### Parkinsonian disability, cognitive status, apathy, treatments and electrode locations 1 and 2 years after surgery

**Fig 4:**
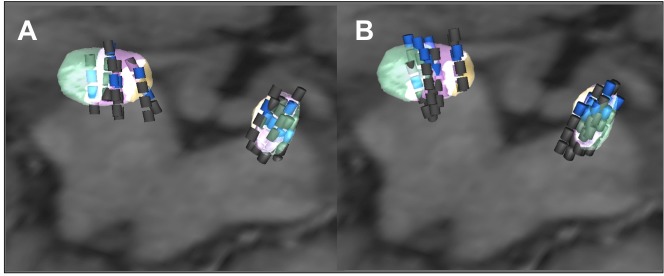
Electrode locations on postoperative MRI in the 17 PD patients operated for STN-DBS. Superior view of the STN after fusion with the three-dimensional MRI acquisition, an axial plane of which is shown. Electrode localisation is presented along the long axis of each electrode with transparent rendering of the three STN territories (limbic, yellow; associative, purple; motor, green). The active contacts are blue and the non active contacts are grey. Using this strategy, the mean mediolateral, anteroposterior and dorsoventral coordinates of active contacts in the 17 PD patients were 11.4±1.5 mm, 9.3±1.4 mm and 3.0±1.7 mm, in the anterior commissure-posterior commissure (AC-PC) space respectively. Panel A shows the electrode locations for the 7 patients who received the perioperative psychoeducation programme. Panel B shows the location of the electrodes in the 10 patients without perioperative psychoeducation programme.

One and two years after surgery, we found no significant differences in the changes in the parkinsonian motor disability, activities of daily living and drug treatments, except for a smaller increase in the parkinsonian motor disability score (UPDRS part III, ‘on’ stimulation ‘on’ drug) (*p* = .011) in patients with psychoeducation *versus* the usual treatment 2 years after surgery (Table 2). After surgery, in the usual treatment group, 8/10 and 6/10 patients had dopaminergic agonists, 1 and 2 years after surgery, respectively, and 4/7 patients in the psychoeducation group both at 1 and 2 years after surgery. No significant differences in electrode locations or in stimulation parameters settings were found between groups (Fig 4).

The changes in cognition (MDRS and FAB scores) and apathy were not significantly different between groups (Table 2) with 1/7 and 2/10 patients developing apathy one year after surgery in the psychoeducation and usual treatment groups, respectively, and 2 patients in both groups 2 years after.

### Carers

The changes in the anxiety (STAI) and burden (ZBI-22) scores between baseline and the first and second year follow-up evaluations did not differ between carer groups (psychoeducation versus usual treatment group). However, carers who followed the psychoeducation programme tended to have a greater anxiety decrease 2 years after surgery compared to carers from the usual treatment group (*p* = .11, Table 2).

### Serious adverse events

In the post-operative period (0–3 months after surgery), 2 patients suffered transient confusion with psychosis which was treated with an antipsychotic drug (clozapine) (P08 and P14), 2 patients presented an acute hypomanic status which disappeared entirely after changes of the stimulation parameters (P02 and P04) and 1 patient had a pulmonary embolism which was treated with an antivitamin K drug (P02). One patient was operated 1 year after surgery for knee prosthesis because of chronic arthritis (P01).

## Discussion

In this randomised controlled study, a perioperative psychoeducation programme was found to prevent postoperative maladjustment in all subdomains, except for one patient, suggesting that it is an effective means of avoiding the social maladjustment paradox in PD patients following STN-DBS.

The postoperative social maladjustment following STN-DBS has been attributed, at least partially, to the “burden of normality” syndrome. This syndrome has been described in patients suffering from severe chronic diseases which benefit from treatment that dramatically improves their clinical status and is characterised by psychological, behavioural and social function disturbance, including intra-couple relationship dynamics [46–49]. It has been identified in patients with chronic illness that suffer a severe disabling condition and the disruption produced by the experience of being “cured”, with disturbed self-image and identity and difficulty in relinquishing the impaired status. The “normal” condition causes a highly demanding pursuit to finally fulfil life events that have been previously missed out on [46]. It is hypothesised as being a psychosocial maladjustment derived from the confrontation of patients’ negative beliefs of their illness and unrealistic expectations which affects the pre/post-treatment transition, although it is understood to stem from the pre-treatment period [46]. This mismatch, produced by patients’ unrealistic or non-specific expectations, has been associated with psychosocial maladjustment during the post-treatment phase of other chronic diseases [18,47,48]. Previous studies have shown that psychoeducation interventions are effective in preventing such post-treatment syndromes in cardiac [22], bariatric [26] or transplant surgeries [23,24] and oncological treatment [25,27,28,50]. However, to our knowledge, no such specific approach has been tested to avoid this maladjustment in PD patients undergoing STN-DBS. The recent identification of social maladjustment in PD patients undergoing STN-DBS led therapists to suggest a multidisciplinary psychosocial preparation of patients and carers [12,17]. Here, we propose a psychoeducation programme that includes educational elements based on the representations and treatment expectations of patients and carers, through the expression of their hopes/beliefs about life changes after surgery. Investigator interventions were focussed not only on providing participants with systematic information but also on performing cognitive corrections to maladaptive thinking patterns, through the ‘Question and Answer’ sessions, allowing for both patients and carers to anticipate physical and emotional changes, whilst paying particular attention to unrealistic/nonspecific expectations. Thereby, the fact that maladjustment was avoided in PD patients included in the psychoeducation group suggests that this perioperative approach is quite specific and effective in avoiding, at least partially, this complex post-operative syndrome.

Maladjustment prevention obtained through our psychoeducation programme in PD patients after STN-DBS may also result from other effects. This could result from the reduction of motor disability and levodopa-related complications as well as an improved quality of life induced by STN-DBS. However, the fact that both post-operative motor and quality of life improvements did not differ between groups does not support this hypothesis. Moreover, maladjustment was previously reported in PD patients with a dramatic improvement of their motor status after STN-DBS [12,51]. Conversely, the aggravation of socio-familial adjustment observed in patients without a psychoeducation programme could result from a smaller decrease in dopaminergic agonist daily dosage and/or the occurrence of a post-operative apathy in these patients, as previously reported [52]. The fact that apathy was similarly reported in both groups with no significant difference in the dopaminergic agonist reduction after STN-DBS is not in line with this hypothesis. Lastly, PD patients whom participated in the psychoeducation programme suffered less anxiety and depression after STN-DBS, with carers showing a similar trend with reduced anxiety. This could suggest that our psychoeducation programme may also have positive effects on anxiety and depression.

Our study has some limitations. First, the small number of subjects included and multiple testing for secondary outcomes prohibits generalisation of the results obtained in this preliminary study. Second, we could not exclude the effects of other psychological or neurological factors in the improved social adjustment after surgery in patients that participated in the psychoeducation programme. Indeed, the fact that psychoeducation was performed by medical doctors and psychotherapists who usually care for these patients in our centre may interfere with the positive impact of the programme *per se*. More specifically, features concerning the relationship between investigators and patients (e.g. the investigators’ therapeutic attitude or empathy towards the patient) were not controlled for. To limit this interaction as far as possible, investigators were asked to pay attention to providing similar care for each patient). The fact that the same therapists followed the patients in the two treatment groups (psychoeducation and usual treatment groups) with no significant difference in the number of outpatient visit, parkinsonian disability, psychotropic or antiparkinsonian medical drug treatments, electrode locations or DBS parameter settings, suggests, at least partly, that the medical care was not significantly different between groups. Third, we did not specifically assess the personality traits of patients and carers, depression and quality of life in carers and their possible changes with STN-DBS, these parameters being identified as potential contributors to the post-operative perceived outcome [13]. This prevents us from precisely assessing the role of such psychological parameters on the effects of our psychoeducation programme. However, except for the hypomanic episodes that occurred in 2 patients after surgery, none of our patients or carers subjectively reported personality changes after surgery. Lastly, the fact that the changes in the burden score were not significantly different between carer groups after surgery suggests that the carers’ perceived medical outcome is quite similar between groups. Finally, the results obtained in this pilot study suggest designing a larger randomised double-blind study with accurate control of psychological factors, in both patients and carers, that could intervene with the postoperative sociofamilial adjustment.

## Conclusions

In conclusion, this preliminary study provides arguments for the importance of preparing PD patients for their post-operative condition, taking into account the social, familial and marital consequences in close relationship with the carer, in order to prevent social maladjustment following STN-DBS. These results need to be confirmed in future larger studies with double-blind assessment, and also to demonstrate its feasibility from an ecological point of view and identify predictive factors.

## Supporting information

S1 CONSORT Checklist (DOC)

S1 File (PDF)
