# Supplementary material for: Tackling psychosocial maladjustment in Parkinson’s disease patients following subthalamic deep-brain stimulation: A randomised clinical trial
Source: PLoS One. 2017 Apr 11;12(4):e0174512. doi: 10.1371/journal.pone.0174512 (PMC5388322; doi:10.1371/journal.pone.0174512)
Supplement: S1 File — (PDF) [file pone.0174512.s002.pdf]

Psychoeducation programme Session 1

Summary

- Surgical procedure
- Post-operative follow-up
- Post-operative expected results

1. Surgical procedure

Stereotactic targeting

The day before surgery

- Stereotactic frame under local anesthesia
- Brain MRI

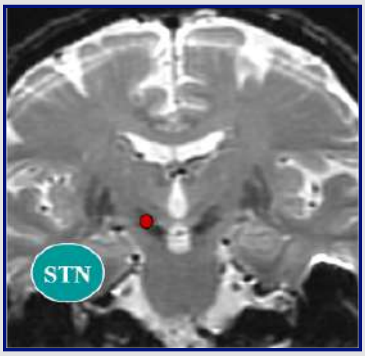

Surgery

Under general anesthesia

Pictures of the drill hole

Stereotactic frame and microelectrode recordings

Pictures of the patient with the frame and microdrive

Neuronal recordings

Pictures of the microdrive on the patient's head

Microelectrode descent

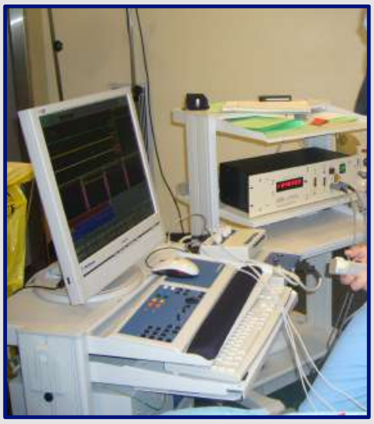

The subthalamic neuronal activity confirms the correct targeting

Neurological testing

Peri-operative stimulation and testing in awake patient

Pictures of the per-operative neurological testing

Clinical effect:

- Rigidity
- Akinesia
- Tremor

Implantation of the definitive electrodes

Best clinical effect

Pictures of the implantation of the definitive electrode

Electrode fixation

Radiological control

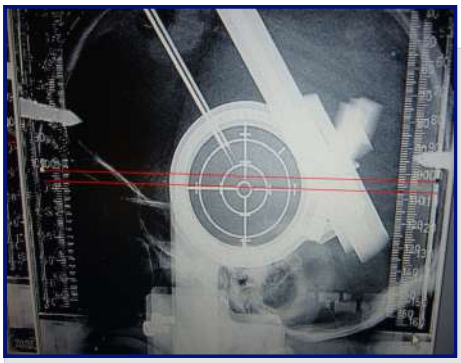

During the microelectrode descent

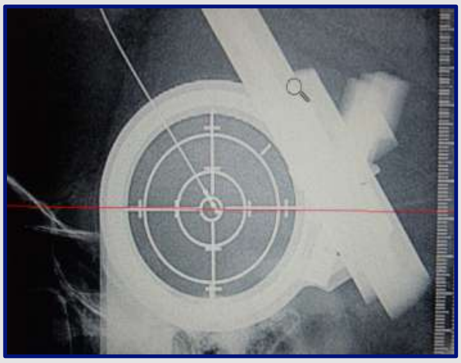

Definitive electrode position

Post-operative radiological control

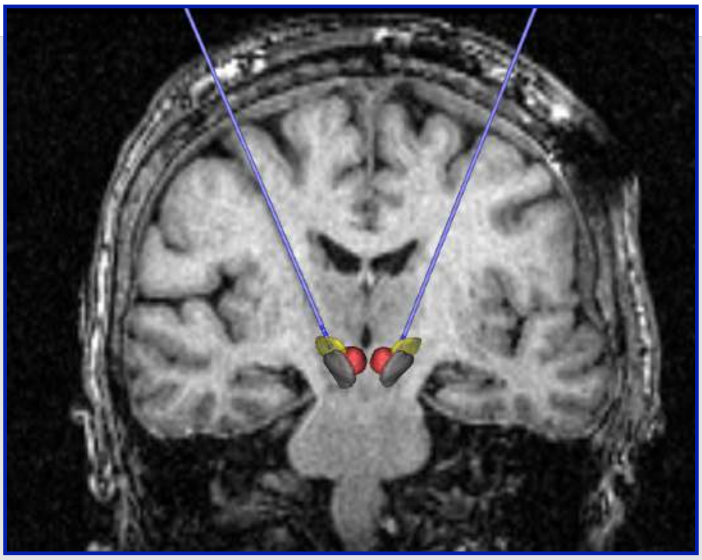

Video of the surgical procedure

Neurostimulator implantation

- Under general anesthesia
  The same day or few days later
- Connected to the electrodes with cables
- Localised within the subclavicular area

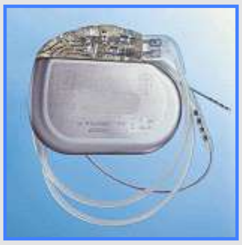

Surgical complications

Rare = 2 to 5%

Usual with (brain) surgery

- Infection (5%)
- Brain Hemorrhage (0,5%)
- Epilepsy

2. Post-operative follow-up

Parameter settings

- During hospitalisation
  - Transient lesioning effect
  - First parameter settings during hospitalisation
  - Concomittant reduction in the medical drug treatment dosage
- Outpatient visit follow-up
  - Progressive adjustment of parameter settings
  - Progressive optimisation of medical drug treatment

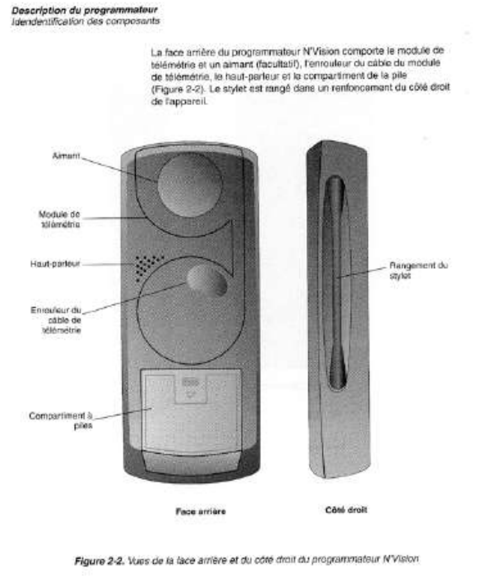

Figure 2-2. Vues de la face arrière et du côté droit du programmateur N'Vision

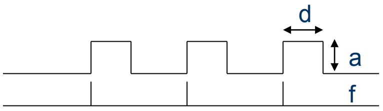

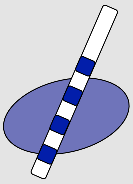

- Contact
- Frequency (f)
- Pulse width (d)
- Amplitude (a)

Motor disability

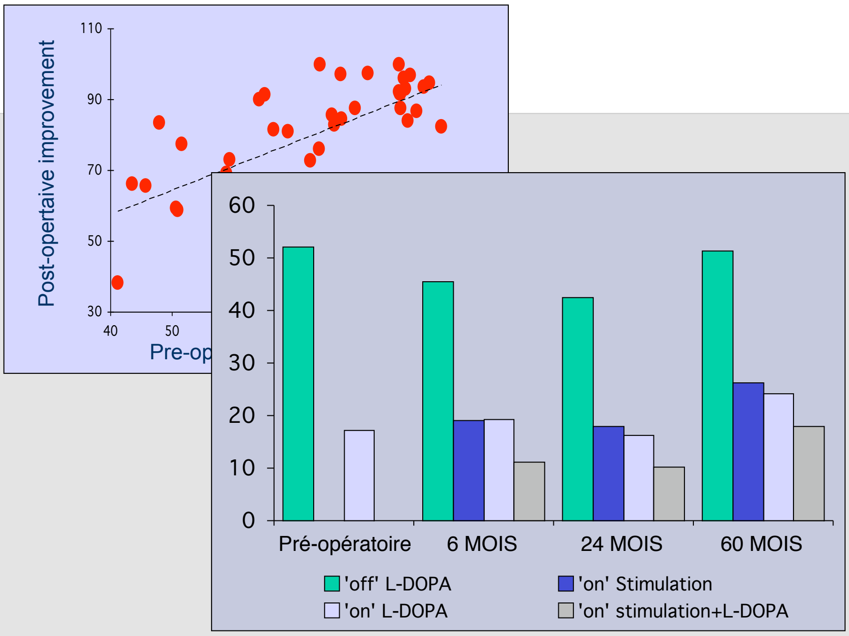

Axial motor signs

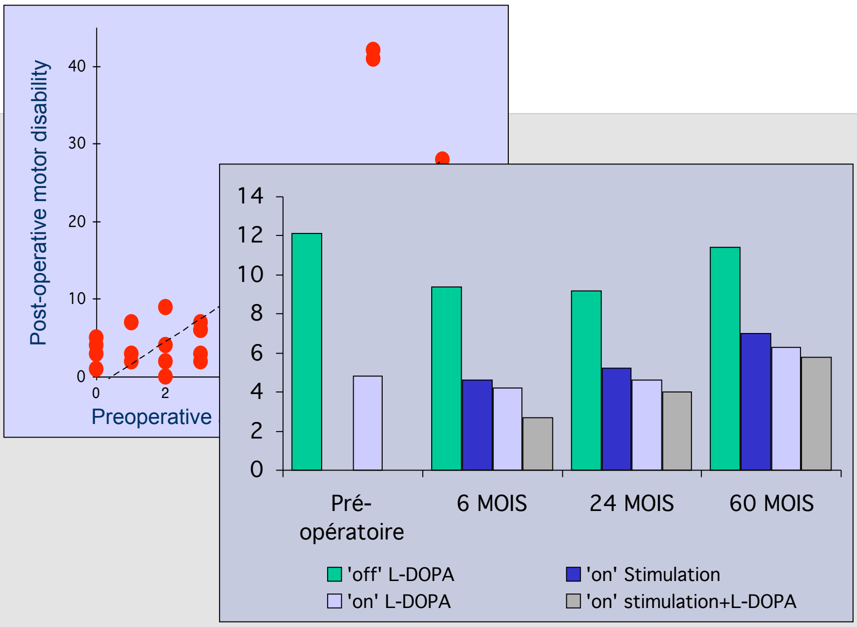

Predictors of good post-operative outcome

1) Young age

2) Excellent response to levodopa treatment

3) No or few axial motor signs unresponsive to levodopa treatment :
gait and balance disorders

4) No cognitive decline

Levodopa related motor complications and drug treatment

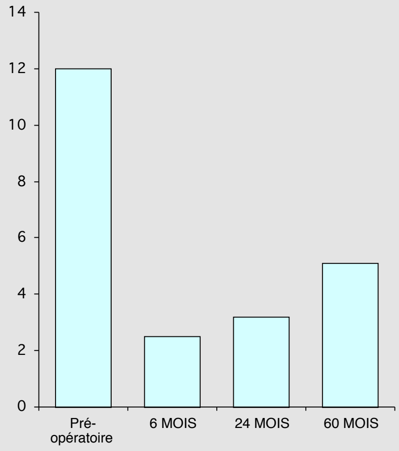

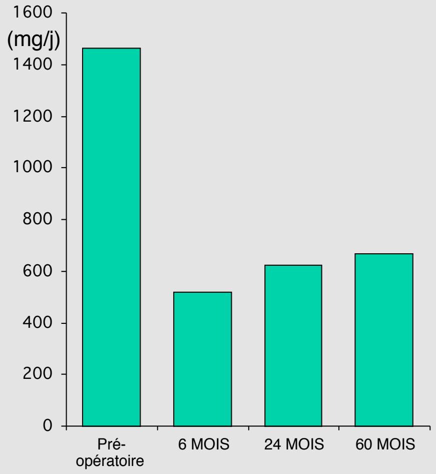

Quality of life

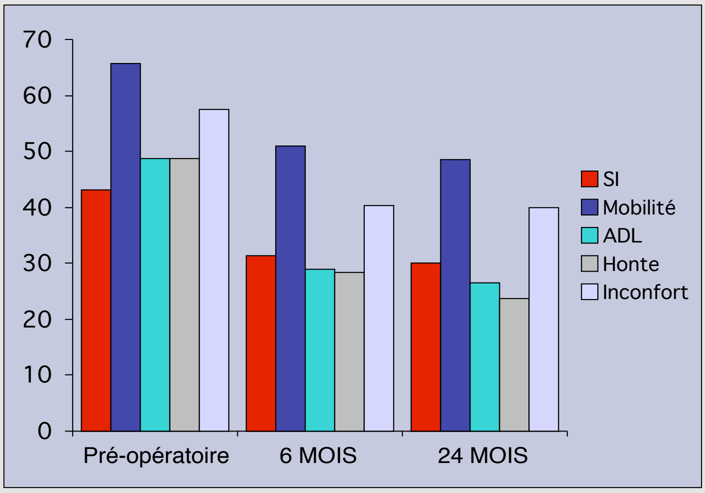

Depression and Anxiety

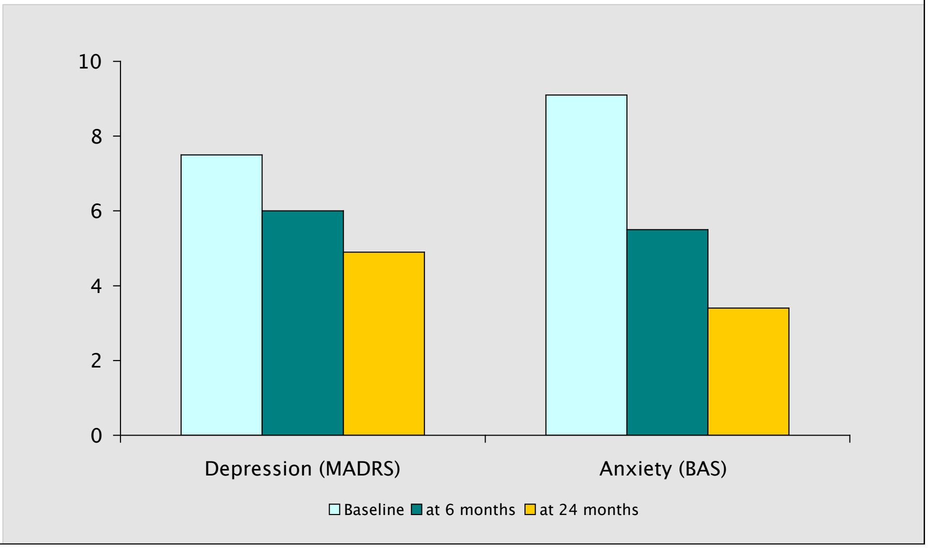

Social adaptation

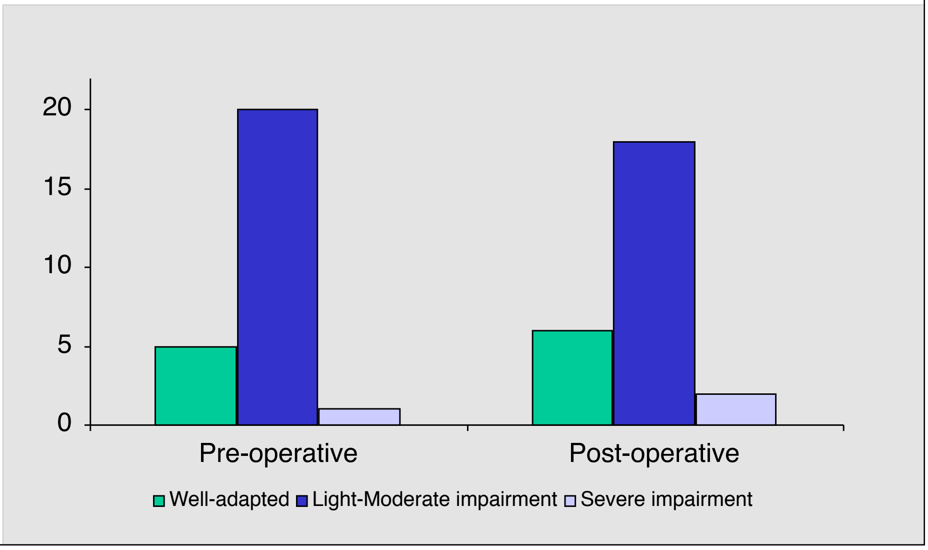

Side-effects

- Weight gain (+ 5kg)
- Eye lid apraxia
- Apathy
- Hypomania
- Dysarthriae
- Rare
  - Psychiatric decompensation
  - Severe dyskinesias

Video of a patient with apathy, and his spouse

Video of a patient with hypomania

Weaknesses

- Aggravation of axial motor signs
  - Freezing of gait and postural instability
  - Dysarthria
  - Swallowing disorders
- No impact on disease progression

Neurostimulator replacement

- Every 5 to 7 years
- During a short hospitalisation
- Under local anesthesia (if possible)

Psychoeducation programme
Sessions 2-3-4

EXPECTATIONS
Social and professional life

REALISTIC	UNREALISTIC

Video of a patient relating social difficulties

Today, having friends is for me…..

Very important | Not important at all

After surgery, having friends will be for me…..

Very important | Not important at all

Today, working is for me…..

Very important | Not important at all

After surgery, working will be for me…..

Very important | Not important at all

EXPECTATIONS
Familial life

REALISTIC	UNREALISTIC

Video of a patient relating familial difficulties

Today, my family life is…..

Easy — Very difficult

After surgery, my family life will be…..

Easy — Very difficult

EXPECTATIONS
Life as a Couple

REALISTIC	UNREALISTIC

Video of a patient and spouse relating couple difficulties

Today, my life as a couple is…..

Serene Conflictual

After surgery, my life as a couple will be…..

Serene Conflictual
